# Normalization of cell associated antiretroviral drug concentrations with a novel RPP30 droplet digital PCR assay

**DOI:** 10.1038/s41598-018-21882-0

**Published:** 2018-02-26

**Authors:** Shetty Ravi Dyavar, Zhen Ye, Siddappa N. Byrareddy, Kimberly K. Scarsi, Lee C. Winchester, Jonathan A. Weinhold, Courtney V. Fletcher, Anthony T. Podany

**Affiliations:** 1 Antiviral Pharmacology Laboratory, Department of Pharmacy Practice, College of Pharmacy (COP), University of Nebraska Medical Center (UNMC), Omaha, NE 68198 USA; 2 Department of Pharmaceutical Sciences, COP, UNMC, Omaha, NE 68198 USA; 3 Department of Pharmacology and Experimental Neuroscience, College of Medicine, UNMC, Omaha, NE 68198 USA

## Abstract

Quantification of antiretroviral (ARV) drug concentrations in peripheral blood mononuclear cells (PBMCs) and tissue isolated mononuclear cells (TIMCs) from lymph node (LNMC) and rectum (RMC) is an important measure of bio-distribution. Normalization of drug concentrations is critical to represent tissue drug concentrations and to analyze both intra-individual and inter-individual variability in drug distribution. However, a molecular method to normalize intracellular drug concentrations in PBMCs and TIMCs methanol extracts is currently unavailable. In this study, a novel droplet digital PCR (ddPCR) assay was designed to amplify RPP30 gene sequence conserved in human and non-human primates (NHP). Genomic DNA (gDNA) isolated from 70 percent methanol embedded PBMCs and TIMCs was used as ddPCR template to quantitate precise RPP30 copies to derive cell counts. The novel molecular method quantitated RPP30 copies in human and rhesus macaque gDNA templates with greater accuracy and precision than qPCR. RPP30 ddPCR derived cell counts are strongly correlated with automated cytometer based cell counts in PBMC (R = 0.90, p = 0.001 and n = 20); LNMC (R = 0.85 p = 0.0001 and n = 22) and RMC (R = 0.92, p = 0.0001 and n = 20) and achieved comparable normalized drug concentrations. Therefore, the RPP30 ddPCR assay is an important normalization method in drug bio-distribution and pharmacokinetic studies in humans and NHPs.

## Introduction

In the current era of antiretroviral therapy (ART), quantification of cell associated drug in peripheral blood mononuclear cells (PBMC) and tissue isolated mononuclear cells (TIMCs) from lymph node (LNMCs) and rectum (RMCs) is a measure of the drug’s cellular and tissue bioavailability and bio-distribution. Normalization of drug concentrations with the total number of TIMCs is critical for standardizing drug concentrations at the pharmacologic site of action and to determine intra- and inter-individual variability of drug bio-distribution. However, precision and accuracy of isolated cell counts is influenced by several factors, including, the presence of cellular aggregates due to cell adhesion, incomplete tissue digestion, red blood cell contamination, decreased cell viability and increased cell death during sample processing. Additionally, differences in cell counting techniques may decrease the overall accuracy of cell counts.

Currently available manual and automated methods to measure cell counts have long processing times and frequently lead to erroneous results due to their lower sensitivity range, which quantify 0.05 to 10 million cells. Additionally, automated cell counters only quantify cells between 2–70 µM in size^1^, yielding the possibility that some types of cells and aggregated cells may not be counted. Recent advances in cell enumeration technologies utilize methods to quantify the total number of nucleated cells by fluorescent 2-(4-Amidinophenyl)-1 H-indole-6-carboxamide (DAPI) staining tandem flow cytometry in automated nucleo-counters such as NC-100 (Chemometec, Allerod, Denmark)^2^. Unfortunately, this method is unable to quantify larger cell aggregates and cannot be applied for high throughput applications due to additional sample processing steps^2^.

Unlike conventional cell counting methods, real-time polymerase chain reaction (RT-PCR) based molecular methods utilize genomic DNA (gDNA) extracted from cells, are highly sensitive and amplify low copy DNA sequences in the human genome, yielding higher specificity and a broader quantification range^3–9^. However, these assays were non-quantitative^3,5–8^ or semi-quantitative^4^ and could not quantify absolute cell numbers. In addition, use of ‘alpha-satellite’ sequence, a tandemly repeated DNA sequence present in the centromeric region of human chromosomes and ‘Alu’ sequence, a short stretch of transposable (mobile) DNA sequence present in several parts of the human genome lead to copy number variability and inconsistent results^6^. Single copy human genomic sequences, located in human down syndrome region of 21^st^ chromosome’s thymidine kinase (TK1) Alu repeat intronic sequences and ribonuclease P (RNase P or RPP30) genomic sequences present on chromosome 10, provide cell counts based on an external reference standard generated from the serial dilution of a highly concentrated plasmid stock in RT-PCR assays^6,10^. However, small changes in reference standard concentrations used in previous RT-PCR methods of RPP30 has led to inaccurate cell enumeration results^11^. Unlike other genomic sequences, only a single copy of RPP30 genomic sequence is present in the human genome. The protein encoded by RPP30 catalyzes the processing of 5′ leader sequences of precursor tRNAs (pre-tRNAs). The RPP30 sequence is immobile and has homologous sequences in both humans and rhesus macaques (RMs) (Macaca mulatta), a widely used animal model of HIV.

Recently available droplet digital PCR (ddPCR) technology partitions a single PCR reaction into many nanoliter sized droplets and amplifies DNA sequences in each droplet to provide an accurate copy number of a single gene sequence without the use of an external reference standard and multiple replicates^12,13^. Technically, in a droplet digital PCR (ddPCR) assay, >20,000 individual monodispersed droplets are generated from each DNA sample and automated droplet generation oil for probes followed by amplification of target template in each droplet utilizing sequence specific primers and fluorescently labeled taqman probes^11^. Following each cycle of DNA polymerization chain reaction (PCR), fluorescent reporters are released from fluorescent dye conjugated oligonucleotide probes and are read by a fluorescent detector as a positive copy of a target sequence. Poisson distribution of data and a probability analysis performed in ddPCR software provides absolute copy numbers of a target gene sequence. Previous studies used RPP30 assay in qPCR platform to determine human genomic DNA extraction efficiency^14^, as an endogenous control to diagnose and quantify the bacterial load in the human genomic DNA background^15^ and as a reference of single copy gene to determine copy number variation of a gene in the human genome^16^. Other studies have used RPP30 assays to normalize copies of infectious agents with cell counts based on the presence of two copies of RPP30 in each cell^17^. However, the direct association of RPP30 copies with cell counts measured in automated cytometer or other cell counting methods was not determined, nor was a validation of the correlation between RPP30 and cells counts shown or referenced. In HIV research, non-human primates (NHPs) are widely used to develop novel antiretroviral drug regimens and immunotherapies. An RPP30 assay that can amplify both human and NHP genomic sequences to determine cell counts would be a useful tool to normalize drug concentrations in pharmacokinetic studies. Herein, we present the details of a novel taqman ddPCR assay designed to amplify human and RM RPP30 sequences from gDNA extracted directly from samples of PBMCs and TIMCs of human as well as RMs collected and stored in 70% methanol for intracellular drug concentration analysis. Since each diploid human cell contains two copies of RPP30 gene, half of the total number of RPP30 copies quantified in ddPCR assay represents cell counts. The assay was validated to determine the precise number of RPP30 copies in gDNA amounts as low as 100 pg to measure 50 mononuclear cells per sample with high accuracy and precision in both human PBMCs and TIMCs. The assay provides a strong correlation to cell numbers attained by automated and manual cytometers for cells isolated from PBMCs, LN and rectum and was useful to normalize antiretroviral drug concentrations in these tissues.

## Results

### Identification of highly conserved RPP30 gene sequences in the genome of human and non-human primates

**Figure 1:**
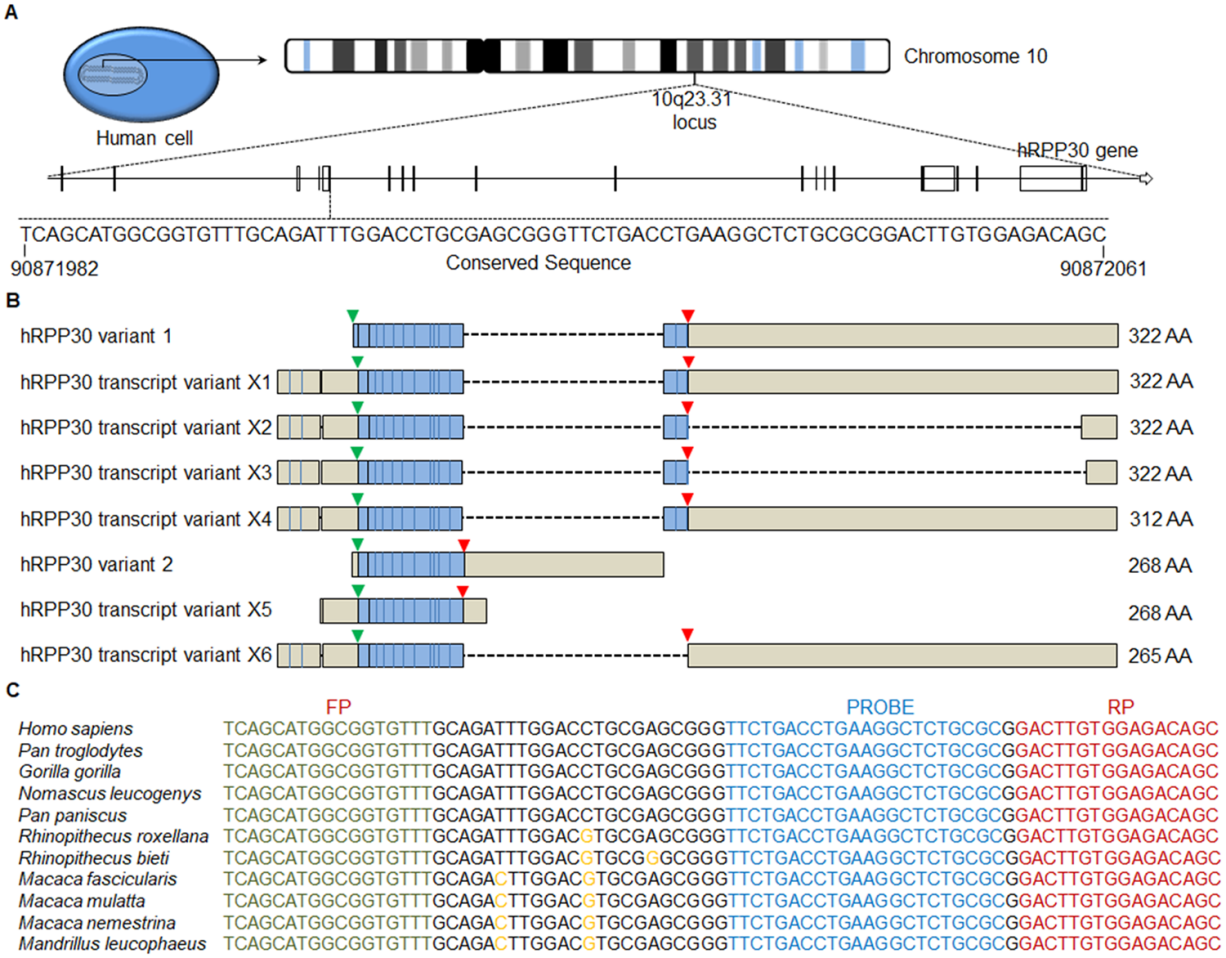
Human RNase P (hRPP30) gene target template sequence (Accession number NC_018921). (**A**) hRPP30 79 base long amplicon spanning five bases in 5′ UTR region and 74 bases in the region (90871987 to 90872071) that encodes a part of the first exon and located at 10q23.31 on the chromosome 10. (**B**) Various domains in human RPP30 mRNA isoforms are shown. Translation initiation site (green) and translation termination site (red) arrows and protein encoding (blue colored) region are shown. (**C**) Clustal Omega multiple sequence alignment analysis of RPP30 amplicon’s genomic sequences in humans and non-human primate species. Forward primer (FP, green), reverse primer (RP, dark red) and probe (blue) conserved sequences and non-homologous mutations (orange) in human and various non-human primate species are shown.

The RPP30 gene encodes a ribonuclease enzyme, which cleaves RNA to synthesize functional small non-coding RNAs. A unique single copy of RPP30 genomic sequence (Accession no# NC_018921) is located at 10q23.31 on chromosome 10 in which a highly conserved sequence spanning 92909573 to 92909633 was identified with the multiple sequence alignment tool in the European Molecular Biology Laboratory at the European Bioinformatics Institute’s Clustal Omega online program (Fig. 1A). The conserved RPP30 gDNA sequence was further selected based on 100% homology among known human mRNA transcripts of the enzyme. The selected RPP30 target sequence overlaps a part of the 5′ untranslated (UTR) region and translation start site in RPP30 mRNA variant 1 and 2 as well as transcript variants X1 through X6 (Fig. 1B). We further selected a RPP30 target genomic sequence that contains >95% sequence similarity among 10 different NHP species (Supplementary Figure S1) that are either natural hosts of immunodeficiency viruses or widely used animal models of HIV infection, including RMs and pig tailed monkeys (Macaca nemestrina) (Fig. 1C).

### Precision and Accuracy of RPP30 droplet digital PCR assay

A novel RPP30 Taqman ddPCR assay that can amplify both human and NHP template sequence was designed based on the selected highly conserved target sequence. The RPP30 ddPCR assay’s primer and probe nucleotide sequences were designed based on similarity in human and NHP genomic sequences. The current RPP30 assay target sequence partially overlaps with previously published target sequence from Luo *et al*.^14^. However, forward and reverse primers used herein have dissimilar sequences so as to account for both NHP genomic sequences (Supplementary Figure S2) as well as human genomic sequences. Experimental methodology steps of the RPP30 ddPCR based cell enumeration method from initial step of gDNA extraction from cell samples (PBMCs and TIMCs in 70% methanol) through quantification of RPP30 copies following a ddPCR work flow to derive cell counts are shown in a schematic diagram (Supplementary Figure S3). To determine the precision of the assay, gDNA samples of 11 different concentrations at two fold increments from 100 to 100000 pg of human, RM and mouse gDNA were used as a template to amplify RPP30 target sequences. Human and RM gDNA templates were readily amplified to provide total number of RPP30 copies. Notably, 100–100000 pg concentration of mouse gDNA did not show any non-specific RPP30 sequence amplification in the ddPCR assay (Supplementary Figure S4) confirming that the RPP30 assay has no background and does not show false positivity while using non-specific templates. To determine the precision and accuracy of the RPP30 ddPCR assay, we quantitated RPP30 copies in 100 to 100000 pg gDNA templates of human and RM reference standards as described in methods. Notably, the novel RPP30 assay quantitated RPP30 copies in as low as 100 pg of human and RM gDNA template in intra, inter-laboratory and inter-operational assays (Supplementary Figure S5A to S5F). Linear regression analysis was performed for the data obtained from intra-laboratory, inter-laboratory and inter-operational RPP30 ddPCR assays. At lowest tested human gDNA concentration (100 pg), we observed <7.6%CV in intra-assay (n = 3) and <10.8% overall %CV in inter-assays of human (n = 8) and RM (n = 9) RPP30 ddPCR assays (Supplementary Tables S1 and S2). Whereas, <12.4%CV in intra-assay and <9.2 overall %CV in inter-assays of human and RM RPP30 ddPCR assays was observed at highest tested gDNA concentration (100000 pg) (Supplementary Tables S1 and S2). These results from human and RM ddPCR assays confirm high precision of the RPP30 ddPCR assay. A highly significant correlation between the amount of human gDNA template with the total number of RPP30 copies was observed in intra-laboratory (R^2^ = 0.996, p < 0.001), inter-laboratory (R^2^ = 0.997, p < 0.001) and inter-operational (R^2^ = 0.994, p < 0.001) RPP30 ddPCR assays (Supplementary Figure S5A to C). In RM gDNA samples, the RPP30 assay performed equally well and results showed significant correlation in intra-laboratory (R^2^ = 0.975, p < 0.001), inter-laboratory (R^2^ = 0.989, p < 0.001) and inter-operational (R^2^ = 0.986, p < 0.001) RPP30 assays (Supplementary Figure S5D to S5F). Residual plots of intra-laboratory, inter-laboratory and inter-operational assays further confirmed low variance (<15% CV) of the RPP30 assays in both human and RM gDNA templates among all tested concentrations of human (Supplementary Figure S5A to S5C, right panel) and RM (Supplementary Figure S5D to S5F, right panel) gDNA templates. One of the major advantages of ddPCR is duplication of the results with greater accuracy and use of less number of replicates^12,13^. We used three replicates for each of the 11 different tested data points with two-fold increase of template concentration from 100 to 100000 pg of human and RM in intra-laboratory, inter-laboratory and inter-operational RPP30 ddPCR assays. A strong correlation was observed between assay derived (observed) RPP30 copies and reference standard (nominal) RPP30 copies in intra-laboratory (R^2^ = 0.986, p < 0.0001), inter-laboratory (R^2^ = 0.999, p < 0.0001) and inter-operational (R^2^ = 0.999, p < 0.0001) human and RM (R^2^ = 0.989, p < 0.0001; R^2^ = 0.993, p < 0.0001; R^2^ = 0.992, p < 0.0001) assays respectively (Supplementary Figure S6A to S6F). These results confirmed high accuracy of the RPP30 ddPCR assay as well as repeatability in different research laboratories and in RPP30 ddPCR assays performed by different operators.

### Precision of quantitative RPP30 real time PCR assays

We used the same series of 11 different human and RM gDNA template concentrations ranging from 100 to 100000 pg to evaluate the precision of qPCR as compared with the ddPCR as demonstrated previously^18^. The cycle threshold (C_T_) values obtained from qRT-PCR experiments were linearized (2^−CT^) to compare with data obtained from ddPCR assays. More than 30.2% CV was observed in all tested gDNA concentrations containing human gDNA template within 100–100000 pg template concentrations. Whereas, higher CV (lower precision) was observed in qPCR assays as compared to ddPCR [<29.2 vs17% CV>] in all tested concentrations of RM gDNA templates showing higher precision of RPP30 ddPCR than qPCR assays (Supplementary Table S3). Notably, qPCR results of two out of six replicates of 200 and 100 pg human gDNA templates, and two replicates of 200 pg and all three replicates of 100 pg of RM gDNA templates showed >35 C_T_ value. In a qPCR assay, samples showing more than 35 C_T_ value have lower confidence due to increased noise to signal ratios^19^ and error rate and should not be considered for further analyses. These results showed lower precision of qPCR than the observed precision of human and RM RPP30 ddPCR assays.

### PBMCs and LNMCs maintain proper cellular size, shape and contain high quality gDNA at increased methanol to aqueous content in drug extraction medium

**Figure 2:**
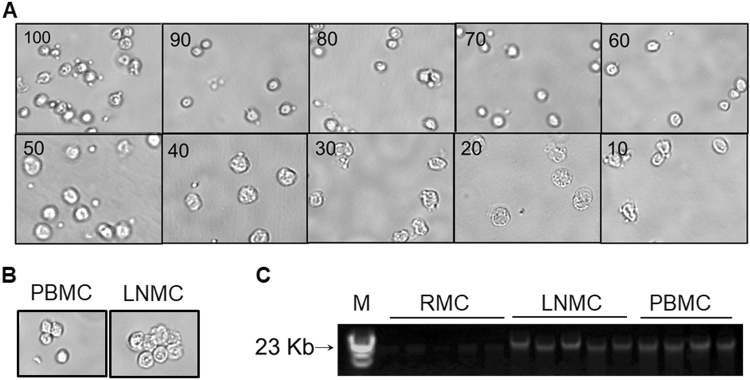
Effect of different percentages of methanol (vol/vol in water) medium used for intracellular drug extraction. EVOS fluid cell microscopic images of (**A**) PBMCs extracted in 10 to 100% of methanol following >60 days storage and (**B**) PBMCs and LNMCs collected from ART treated HIV-1 infected patients in 70% methanol following >6 months storage. (**C**) Qualitative analysis of genomic DNA extracted from mononuclear cells isolated from rectum (RMCs), lymph node (LNMCs) and peripheral blood (PBMCs) of ART patients on one percent agarose gel. M, molecular marker. A part of the gel with gDNA bands was shown.

Prior to utilizing the methanol extracted PBMCs and LNMCs to extract gDNA templates for the RPP30 ddPCR assay and other downstream processes, we examined cellular morphology, analyzed the quality of gDNA and quantitated the amount of drug released into the 70 percent methanol, the most widely used drug-extracting medium to analyze intracellular drug concentrations, with and without lysing the PBMCs. Though cellular morphology and integrity of nucleus in methanol fixative is well described^20^, how cell morphology is influenced in different percentages of methanol used as drug extracting medium is currently unknown. We observed cellular morphology of isolated PBMCs stored in varying methanol composition under the EVOS fluid cell imaging station. PBMCs present in 60 to 100% methanol maintained cellular shape and size as shown in Fig. 2A. Cells stored in 10 to 50% methanol, however, froze due to high aqueous content resulting in loss of membrane integrity. Additionally, cells stored in 10 to 50% methanol showed increased size and granularity as compared with cells stored in >60% methanol. To further confirm our results, morphologies of PBMCs and LNMCs collected from HIV-1 infected patients receiving ART that were stored in 70% methanol for more than one year were examined under the light microscope. Consistent with above results, the morphology of MNCs from treated patients was intact and not influenced by their long-term storage in 70% methanol. (Fig. 2B). Following confirmation of the integrity of cells in methanol extracts, we extracted gDNA from PBMCs, LNMCs and RMCs pelleted from methanol extracted drug samples by high salt extraction of proteins method. Quality of the gDNA was analyzed by agarose gel electrophoresis as described in methods section. Figure 2C and Supplementary Figure S7 show the quality of gDNA isolated from all tissues depicted DNA bands of >23 Kb on agarose gel (Fig. 2C). Moreover, the A_260_/A_280_ ratio of PBMCs and TIMCs derived gDNA measured by the nanodrop instrument in almost all samples was 1.8 to 1.9, which revealed the high purity of gDNA without any protein or RNA contamination. These results confirmed that gDNA extracted from methanol extracted drug samples by the high salting out of proteins method is of high purity and of high quality.

### Non-lysed PBMCs release total amounts of intracellular drugs into the drug extracting methanol medium

ARVs’ release from intracellular compartments into the 70% methanol is currently unknown. We analyzed FTC-TP and TFV-DP in PBMCs of ART treated HIV-1 infected patients in both non-lysed (non-destructed) and lysed (destructed) cells present in 70% methanol fractions. Visual inspection of sonicated PBMCs by EVOS fluid cell imaging station depicted a disrupted cellular morphology, indicative of complete lysis, after the sonication (Supplementary Figure S8). Analysis of cell free lysates with and without prior sonication of the samples from two different ART treated patients for intracellular FTC-TP and TFV-DP concentrations by LC/MS/MS showed similar intracellular drug concentrations (Supplementary Figure S9A and 9B). These data showed that ARVs are readily released into the methanol medium as both lysed and unlysed PBMCs in methanol extracts resulted in similar ARV concentrations.

### Quantitation of PBMCs and TIMCs counts in human tissues using RPP30 ddPCR

**Figure 3:**
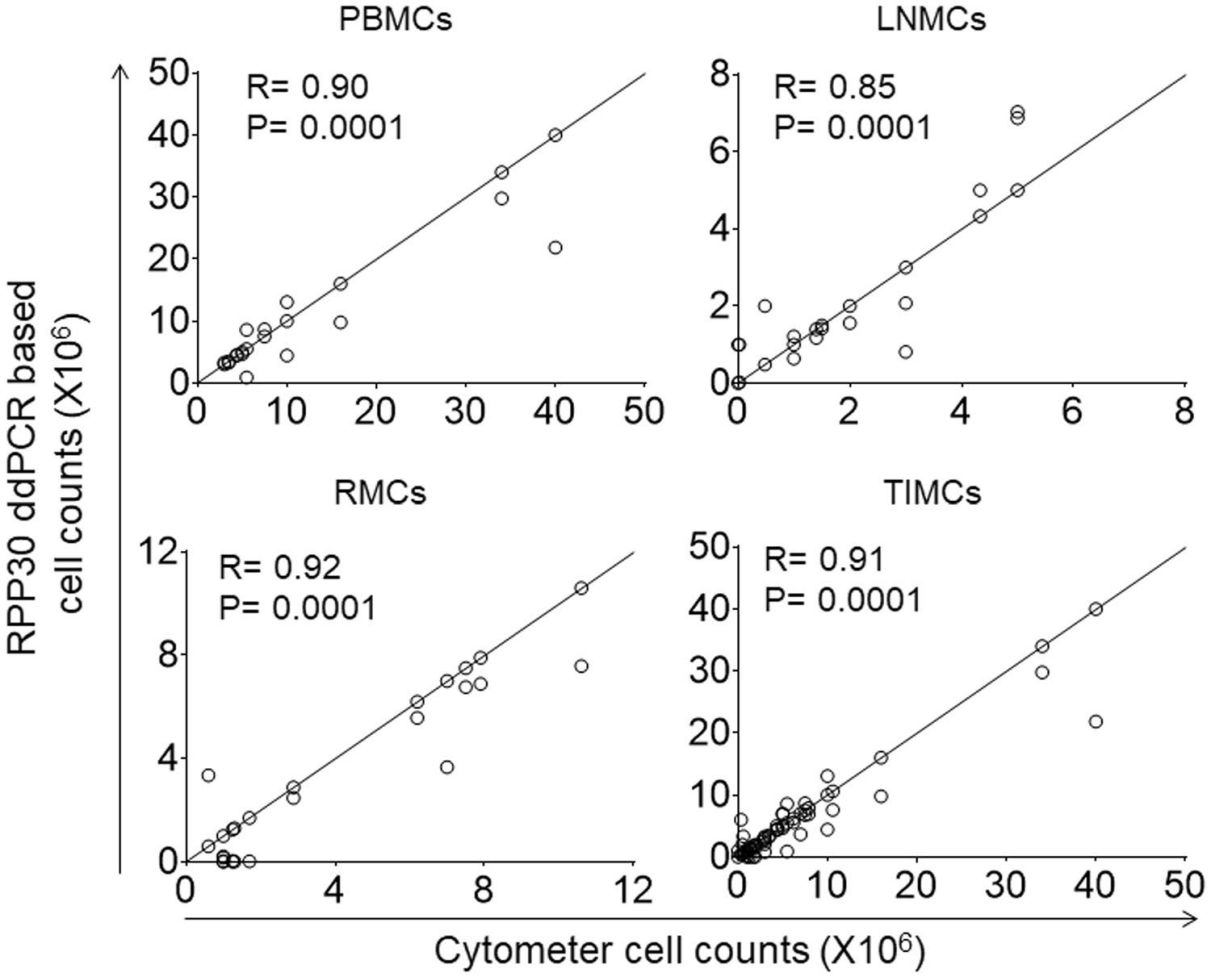
Correlation between cell counts measured by automated cytometer and derived from RPP30 copies quantitated in ddPCR assays. PBMCs, LNMCs and RMCs counts measured by automated cytometer (x axis) and derived from RPP30 copies quantitated in a ddPCR assay (y axis) were shown. Pearson correlation (R^2^) and statistical significance (p value) was analyzed by prism software.

Cell counts were calculated from human PBMCs (n = 20) and TIMCs derived from lymph node (n = 22) and rectal tissue (n = 20) after quantitating RPP30 copies in ddPCR assay. Comparison of the total cell counts derived from the RPP30 ddPCR assay and automated cytometer showed a strong and significant correlation for PBMCs (R = 0.90; p = 0.0001), LNMCs (R = 0.85; p = 0.0001) and RMCs (R = 0.92; p = 0.0001) (Fig. 3). Combined correlation analysis of PBMCs and TIMCs, including, LNMC and RMC samples showed strong linear correlation with high significance (R value of 0.91, p = 0.0001) (Fig. 3). These results confirm that the cell numbers quantitated by RPP30 ddPCR assay are precise and strongly correlated with automated cell counters.

### Normalization of emtricitabine antiretroviral drug concentration with PBMCs and TIMCs counts measured in an automated cytometer and RPP30 ddPCR assays

**Figure 4:**
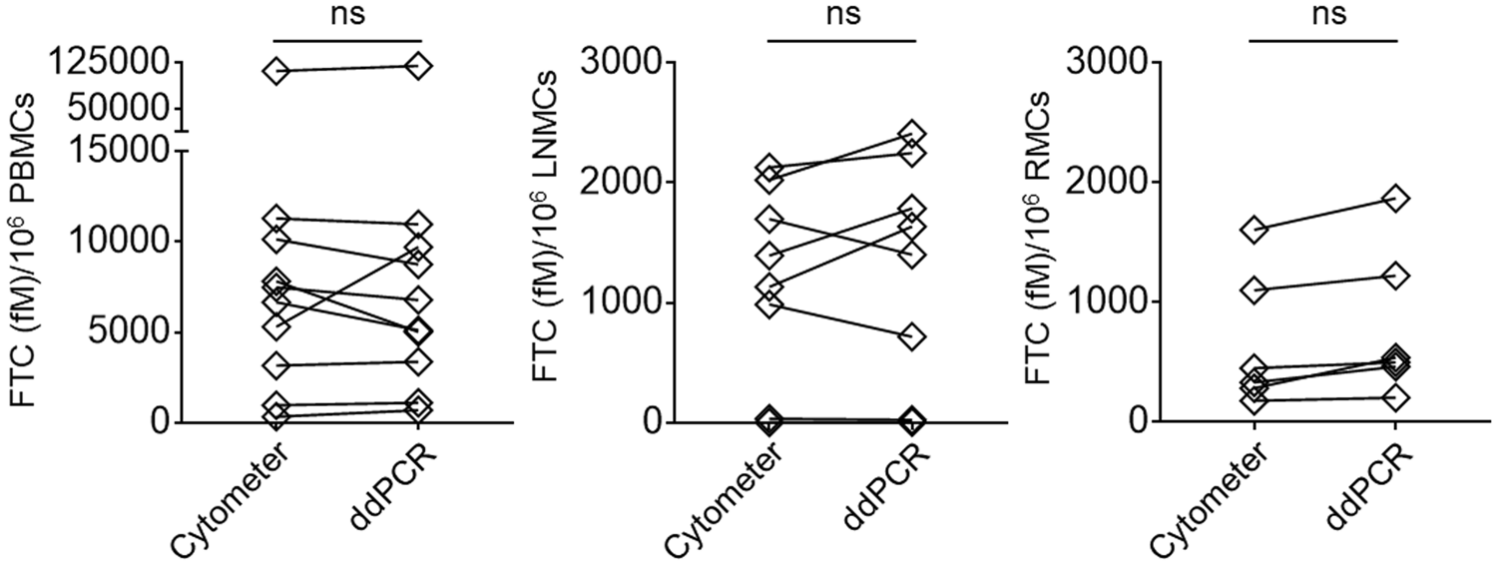
FTC-TP antiretroviral drug concentrations normalized with cell counts measured by automated cytometer or derived from the RPP30 ddPCR assay in human (A) peripheral blood mononuclear cells (PBMC), 4B) lymph node derived mononuclear cells (LNMC) and (C) rectum derived mononuclear cells (RMC) were shown. P values were calculated by t test in Prism software.

Normalization of intracellular ARV drug concentrations quantitated by mass spectrometric analysis from PBMCs and TIMCs is necessary to compare intra-individual and inter-individual bioavailability in different tissue compartments of drug treated patients and animal models. We normalized the concentrations of FTC-TP in human PBMCs, LNMCs and RMCs using the cell counts measured by automated cytometer or the cell counts derived from RPP30 ddPCR assays. We did not identify significant differences between the intracellular FTC-TP concentrations obtained following normalization with automated cytometer based cell counts or using the ddPCR assay derived cell counts in PBMCs (Fig. 4A), and LNMCs (Fig. 4B) and RMCs (Fig. 4C). These results showed that the RPP30 ddPCR assay derived cell counts were comparable in PBMCs, LNMCs and RMCs to the automated cytometer based cell counts. Our compliance with the Standards for Reporting Diagnostic accuracy measures is presented in Supplementary Table S4.

## Discussion

We set three important criteria for developing a novel molecular method of cell enumeration. First, the use of a stable (immobile) gene sequence with constant copy numbers present in the genome of every human and RM cell as a target template. Previous studies have reported non-quantitative^3,5–8^ cell enumeration methods that help in detection of human cells in tissues of SCID or NOD/SCID humanized mice and semi quantitative^4^ or quantitative but not well characterized^17^ cell enumeration methods. The RPP30 ddPCR assay used in our study utilized a stable (immobile) and highly conserved two copies of RPP30 gene sequence present on 10^th^ chromosome in each human cell and on 11^th^ chromosome in each RM cell (Fig. 1A) to quantify absolute RPP30 copies in human and RM gDNA templates (Supplementary Figure S5A to S5F) to derive cell numbers. Second, quantification of absolute copy numbers of the target DNA sequence without the use of external reference standards. Previously reported non-quantitative and semi-quantitative assays utilized real time PCR platform that mandates use of an external reference standard for copy number calibration. The differences in PCR efficiency can have a significant impact on the external reference standard based quantitation and lead to erroneous results^21^. In a study, RPP30 ddPCR assay utilizing droplet digital PCR platform to directly quantify absolute copy numbers of a RPP30 target DNA sequence was used to derive human cell counts. However, this assay merely relied on the RPP30 copies and was not well characterized. Our study’s RPP30 assay quantitated RPP30 copies, comparable to nominal RPP30 copies of human and RM reference standards (Supplementary Figure S6A to S6F) and were also strongly correlated with automated cytometer based human cell counts (Fig. 3). Third, determination of precise and accurate copy numbers using a ddPCR assay to enumerate PBMCs and TIMCs collected for purposes of quantifying cell associated ARV concentrations. This novel cell enumeration assay can count cells with a high degree of precision and provides results congruent with automated cell counters (Fig. 3). Both automated cytometer derived and ddPCR assay based cell counts achieved comparable normalized ARV concentrations (Fig. 4).

We showed that gDNA extracted from PBMCs and TIMCs present in methanolic stored samples is a suitable template for the RPP30 ddPCR assay to derive cell counts. The methanolic storage solvent is ideal for cellular samples because it is compatible with −80 °C long-term storage, providing stability for nearly all currently available ARVs. Additionally, storage media containing high percentages of methanol are highly adaptable to downstream extraction and analytical techniques, whether it is spectrophotometric or LC/MS/MS based detection. Extraction of the total amount of gDNA from methanol embedded TIMCs samples without any loss of the drug is challenging, yet was overcome by directly suspending the cells in 70% methanol for drug concentration analysis and use of high salt extraction of proteins method of gDNA extraction. Previous studies have used methanol evaporation from TIMCs stored samples to extract gDNA^22^ or centrifugation to separate cells from methanol storage media^23^. Though several previous studies successfully utilized the high salting out method for gDNA extraction, column based methods replaced these methods because of their simple methodology^24^. The major drawback of column based methods is that they limit the amount of gDNA bound to the column and are sensitive to numerous factors including salt concentration and detergents in buffers^25–27^. To extract the total amount of gDNA without much interference of cellular materials^25^, we adapted the high salt extraction method^28^. This method can efficiently extract the total amount of gDNA in 70% methanol stored samples with little variability in gDNA recovery as is shown by congruent RRP30 copies in multiple sample aliquots.

The major advantage of ddPCR based enumeration is the prospect of reducing the noise observed in qPCR based assays while eliminating the need for an external DNA reference standard. We used eleven different concentrations ranging from 100 to 100000 pg of human and RM gDNA reference standard samples with known RPP30 copies (15000 copies/50000 pg) to determine the accuracy and precision of the RPP30 ddPCR assay. The total number of RPP30 copies in both human and RM samples were comparable, and a statistically strong correlation was observed between gDNA concentration and RPP30 copies of human (Supplementary Figure S5A and S5C) and RM (Supplementary Figure S5D and S5F) intra-laboratory, inter-laboratory and inter-operational RPP30 ddPCR assays. In addition, a strong correlation was also observed between nominal and observed RPP30 copies of human (Supplementary Figure S6A to S6C) and RM (Supplementary Figure S6D to S6F) intra-laboratory, inter-laboratory and inter-operational RPP30 ddPCR assays. Importantly, these experimental results demonstrated the accuracy and repeatability of RPP30 ddPCR assay in different research facilities performed by different laboratory personnel. Consistent with previous reports, there was no significant difference in copy numbers between three replicates at various tested concentrations^12^, which removes the need of multiple replicates to assay large numbers of samples to estimate RPP30 copies in high throughput formats. We have used series of dilutions of the initial concentration (100000 pg) to evaluate the accuracy of the method as described previously. Our measurements of qPCR and ddPCR assay performance in both linear two fold increments of gDNA template concentration ranging from 100 to 100000 pg in triplicates using the same RPP30 primers and taqman probe validated existing reports^12^. In a qPCR assay, calculated CV for linearized C_T_ (2^−CT^) values (n = 3) of 100 to 100000 pg of gDNA templates showed higher CV over ddPCR assays [<17 vs >29% CV], which indicates greater precision of RPP30 ddPCR assay. We also derived relative amplification efficiency of qPCR (per cycle efficiency with the use of C_T_ method) to determine the accuracy of the assay. The end point reading obtained by ddPCR assays showed higher efficiency over qPCR (per C_T_ efficiency) at various tested template concentrations (100 to 100000 pg) (Supplementary Tables S1 and S2). These data vastly improve on the precision of RPP30 ddPCR assay derived cell enumeration.

ddPCR has close similarity to qPCR in thermocycling steps; however, most of the previous studies utilized >1 min for amplification of target sequence since gDNA is a larger template (>23 Kb) and down-stream applications such as quantification of HIV DNA utilizes digested fraction of human gDNA to amplify target DNA sequences. In our study, we standardized the various steps in thermocycling and 40 cycles of denaturation at 95 °C for 15 sec tandem primer-probe annealing at 55 °C (30 sec and sequence amplification at 72 °C for 30 sec and was completed in 2 hours. This method emphasizes the fact that undigested larger DNA templates (>20 kb) such as human and RM gDNA amplified efficiently following generation of undisrupted droplets containing intact gDNA templates in automated ddPCR droplet generator without any interference in gene copy measurements. We did not use a high amount of gDNA template as the total amount of gDNA extracted from PBMCs and TIMCs was always diluted to utilize small amounts (1000 to 100000 pg) in the ddPCR application. However, 100000 pg of gDNA template showed RPP30 copies below the saturation limit (≥2000 copies/µL reaction) of the assay. Since every gDNA template has a single copy of RPP30 target sequence, the amount of starting material ranging 100 to 1000000 pg is sufficient to derive cell counts in each assay. Our data from PBMCs, LNMCs and RMCs counts demonstrated that samples with lower (≤0.1 million) and higher (≥40 million) cell counts are accurately measured by the RPP30 ddPCR assay using 100 to 100000 pg of gDNA as RPP30 ddPCR template.

In drug concentration normalization experiments, FTC-TP concentration normalized with cell counts measured by automated cytometer and RPP30 ddPCR assays were not significantly different in PBMCs, LNMCs and RMCs. The RPP30 ddPCR assay was found to be highly accurate with higher precision for measuring RPP30 copies in human and RM samples. Moreover, this single RPP30 ddPCR assay accurately counts PBMCs and TIMCs in methanolic stored samples of ART treated HIV-1 infected patients and NHP models of HIV-1 infection. This RPP30 ddPCR method may have several applications including, enumeration of human cells in humanized severe combined immunodeficiency (Hu-SCID) mice models and human xenograft mice models and finding copy numbers of a specific gene in the genome of human and NHP cells. Our primary application was to normalize intracellular concentrations of ARVs, more accurately and efficiently, to improve knowledge of metrics of bioavailability and other pharmacokinetic characteristics for ART studies in HIV-1 infected humans and SIV-infected RMs. For this application, the RPP30 ddPCR assay was a significant advance over existing methods and thus, a useful technique in HIV pharmacology.

## Methods

### Subjects

PBMCs and LNMCs were collected from HIV-1 infected persons participating in a study of antiretroviral therapy (ART) performed at the University of Minnesota as we have described^29^. The study was approved by the Institutional Review Board (IRB) of the University of Minnesota and all subjects gave written informed consent to participate in the study, and to all described procedures that follow. The subjects were enrolled into a clinical protocol where treatment was started after obtaining peripheral blood by venipuncture, inguinal lymph nodes (LN) by excisional biopsy, ileum and rectal biopsies through colonoscopy^29^. In all subjects, peripheral blood, LN, ileum and rectum biopsies were obtained at 3 and 6 months post antiretroviral therapy for quantification of intracellular ARV concentrations. Laboratory procedures for tissue management were described elsewhere^29,30^. Laboratory studies on de-identified samples from these participants, including the use of PBMCs, LN and gut derived tissue isolated mononuclear cells to quantify antiretroviral drug concentrations, and associated studies of cellular morphology and cell enumeration were performed at University of Nebraska Medical Center (UNMC). All methods were performed in accordance with approved guidelines and IRB regulations.

### Ethics Statement for Rhesus Macaque Samples

RMs were housed at UNMC and were cared for under the guidelines established by the Animal Welfare Act and the NIH “Guide for the Care and Use of Laboratory Animals” using protocols approved by the UNMC Institutional Animal Care and Use Committee. All methods were performed in accordance with approved guidelines and regulations.

### Cell Enumeration of Human Derived Cells

PBMCs and TIMCs, including, LNMCs and RMCs were collected from HIV-1 infected persons. For PBMC isolation, blood was collected in acid citrate dextran tubes and processed as previously described^31,32^. Following resuspension of PBMC pellet in RPMI medium, a small 10 µL aliquot of cells was added to 90 µL of trypan blue for counting the cells by hemocytometer and to manually estimate the total number of isolated cells. Additionally, an aliquot of cellular suspension was used for cell enumeration via a Countess II cytometer (Invitrogen Corp, Carlsbad, CA). The cells were next suspended in 70% methanol for storage at −80 °C. LNMCs were isolated from LN tissue biopsies as previously described^32^. An aliquot of cells was suspended in PBS for automated and manual cell counting, while the remainder was suspended in 70% methanol for storage and freezing at −80 °C. Rectal biopsies were processed to prepare rectum derived mononuclear cells as previously described^29^. Rectal MNCs were counted by automated techniques with Countess II cytometer respectively then stored in 70% methanol at −80 °C.

### Cell enumeration of Animal Derived Cells

Rhesus macaque PBMCs were collected from whole blood as per the standard ficoll-paque separation procedure as previously described^33,34^. Macaque derived PBMCs were counted both manually and with automated cell counting techniques [Countess II cytometer (Invitrogen Corp, Carlsbad, CA)] prior to storage in 70% methanol at −80 °C.

### *In-vitro* cell culture

SVEC4-10, a mouse lymphoid endothelial cell (LEC) line was purchased from American Type Culture Collection (ATCC, Manassas, VA) and was grown in Dulbecco’s Minimum Essential Medium (DMEM) supplemented with 10% FBS (ATCC, Manassas, VA) while maintained at 37 °C with 5% CO_2_. Automated counting of SVEC4-10 cells was performed with a Countess II cytometer (Invitrogen Corp, Carlsbad, CA).

### Cell imaging in EVOS Fluid Cell Imaging Station

PBMCs were isolated from the blood of healthy human donors at UNMC Elutriation Core facility. PBMCs were suspended in varying compositions of methanol (10 to 100% methanol solvent with water (vol/vol)). PBMCs and LNMCs collected from patients receiving ART were permeabilized in 70% methanol and were stored for more than one year at −80 °C. Following storage, we examined the morphology of cells under the microscope. Images of the cells were captured at 20X (fixed fluorite objective lens) magnification with a high sensitivity interline charge coupled device (CCD) monochrome camera fixed in EVOS FLOID (Life Technologies, Chicago, USA) cell imaging station. All images were processed and stored in JPEG file formats.

### Cell Associated Drug Quantitation

MNCs stored at −80 °C for >6 months were thawed and analyzed for quantitation of emtricitabine-triphosphate (FTC-TP) as previously described^29,35,36^. Thawed samples were processed by standard methods, applying a solid phase extraction of the sample followed by indirect analysis via LC/MS/MS.

### Genomic DNA Extraction

Isolated MNCs suspended in 70% methanol and a monolayer of SVEC4-10 cells were used as source material for gDNA extracted with modified high salt-extraction of proteins method^28^ to isolate gDNA. Briefly, PBMCs and TIMCs were centrifuged at 1500 rpm for 10 min followed by a round of 5000 rpm for 10 min and the pellet was suspended in 0.6 mL of resuspension buffer [10 mM tris-HCL, pH 8.0 (CAT# BP1758, Fisher Scientific, USA), 0.4 M NaCL (CAT# S3014, Sigma Life Sciences, St. Louis, USA) and 2 mM EDTA (CAT# 03690, Sigma Aldrich, St. Louis, USA)]. Proteins and RNA were digested with digestion buffer [7.5 µL of 10 mg/ml RNase A (0.1 mg/mL) (CAT# 19101, Qiagen Inc, Illinois, USA) and proteinase K (0.25 mg/mL) (CAT# 19131, Qiagen Inc, Illinois) and sodium dodecyl sulphate (SDS) (0.5%) (Sigma Life Sciences, St. Louis)] and incubated at 55 °C for a minimum of 2 h up to 18 h or overnight. Next, proteins were precipitated with 200 µL of 6 M NaCl (1.5 M) and samples were centrifuged at 10,000 rpm for 10 min to remove protein and RNA. gDNA present in supernatant was then precipitated with addition of twice the sample volume of 95% ethanol and centrifuged at 14,000 rpm for 20 min and washed with 0.5 mL of 70% ethanol. Last, the DNA pellet was suspended in TE buffer (10 mM Tris-HCl and 1 mM EDTA) and stored at 4 °C for use within a week or at −80 °C for long term use.

### Agarose Gel Electrophoresis

A 1% agarose gel was prepared by dissolving agarose (Cat# A9539-10G, Thermo Scientific, Dallas, USA) in 1× TAE (40 mM Tris-acetate and 1 mM EDTA, pH 8.3) buffer (Cat# 15558042, Thermo Fisher Scientific, Chicago, USA) and boiled in a microwave. After cooling to room temperature, 0.5 µg/mL of ethidium bromide (Invitrogen, Chicago, USA) was added and poured on a gel cast apparatus (Biorad, USA) set with a 10 well comb. DNA samples were prepared by adding 50 to 100 ng of extracted genomic DNA, 1 µL of 10× Blue Juice Gel loading buffer (Life Technologies, Chicago, USA) and 1× TAE buffer to make up the total volume to 10 µL. DNA samples and 0.5 µg of Lambda DNA Hind III digest (New England Biolabs, Boston, USA) were loaded in separate wells of the 1% agarose gel and was run at 80 V in mini-sub cell GT cell mini agarose gel apparatus (Cat#1704467EDU, Biorad, Los Angeles, USA) containing 1× TAE running buffer. Images of the agarose gels were analyzed in Biorad Quantity One 1-D Analysis Software (Biorad, Los Angeles, USA) as previously described^37^.

### Design of RPP30 ddPCR Probes and Primers

A novel RPP30 taqman ddPCR assay was designed based on the unique and single copy RPP30 gDNA sequence located at 10q23.31 locus on chromosome 10 in each haploid human genome (Fig. 1A) and two copies in a diploid human cell. The RPP30 genomic sequence is present at the NC_018921 locus on chromosome 10, alternate assembly CHM1_1.1 between 92909573 to 92909633 of human whole genome shot gun sequence (Accession no: NC_018921). This sequence is also conserved among genomic DNA sequences of various NHP species and was selected with the multiple sequence alignment tool in European Molecular Biology Laboratory (EMBL) at the European Bioinformatics Institute’s Clustal Omega online program. Probe (5′-VIC-TTCTGACCTGAAGGCTCTGCGC-MGBNFQ-3′), forward (5′-TCAGCATGGCGGTGTTT-3′) and reverse (5′-GCTGTCTCCACAAGTC-3′) primers’ nucleotide sequences with 100% homology among various monkey species was selected with Integrated DNA Technologies Primer Quest tool (Integrated DNA Technologies (IDT), Chicago, USA) and 20X RPP30 ddPCR assay was custom synthesized at Life Technologies coroporation, Chicago, USA. Thermocycling was performed in 4 different steps, initial one step of denaturation at 95 °C (5 min), followed by 40 cycles of sequential denaturation at 95 °C (15 sec), annealing at 55 °C (30 sec) and sequence amplification at 72 °C (30 sec) followed by a single extension step at 72 °C (5 min) and the completed PCR reactions were kept on a final hold at 4 °C.

### Quantitation of RPP30 copies using ddPCR and deriving cell counts

RPP30 copies were quantitated with a RPP30 taqman ddPCR assay using the VIC labeled probe, forward and reverse primers as described above. A QX200 ddPCR system (Biorad, Los Angeles, USA) was used to quantitate RPP30 copies in human and RM gDNA samples according to the manufacturer’s protocol. Briefly, 25 µL of total reaction mixture containing 6.25 µL of gDNA sample (100 to 100000pg of human or RM gDNA), 12.5 µL of 2× Biorad ddPCR super mix for probes (Biorad, Los Angeles, USA), 1.25 µL of 20× RPP30 taqman assay containing forward, reverse primers and probe and 5 µL of nuclease free water was loaded into a 96 well PCR plate (Biorad, Los Angeles, USA). The 96 well PCR plate was sealed with a pierceable sealer and vortexed to mix the reaction followed by centrifuging at 2000 rpm for 2 min. The PCR plate was placed in automated droplet generator (Biorad, Los Angeles, USA) to utilize automated droplet generation oil and ddPCR reaction mixture containing gDNA templates to generate droplets containing gDNA assay templates. An automated droplet generator injected 20 µL of reaction mixture into each well of a DG8 cartridge to generate droplets through mixing droplet generation oil for probes (Biorad, Los Angeles, USA). The generated droplets were collected into a new 96 PCR well plate. PCR thermocycling was performed for the reaction samples containing more than 20,000 droplets with the above described PCR cycle conditions. Following the PCR thermocycling reaction, droplets were read for fluorescence signal positivity in QX200 droplet reader (Biorad, Los Angeles, USA). RPP30 gene copies per total assay volume (20 µL) were quantitated from raw data by Quantasoft Pro software (Biorad, Los Angeles, USA). Half of the total number of RPP30 copies equated cell numbers from the original samples. To compare inter-instrument ddPCR performance, RPP30 copies were also quantitated in select human clinical samples using Quant studio (QS) 3D. The QS ddPCR assay reaction was performed in 14.5 µL total volume containing 1.4 µL of genomic DNA per sample as a template (100–50000 pg), 7 µl of 2× QS 3D v2 master mix (Life Technologies, Chicago, USA), 0.7 µL of 20× RPP30 assay, 5.4 µL of nuclease free water and was loaded on to the high-density QS digital PCR v2 chip (Life Technologies, Chicago, USA) containing 20,000 reaction wells on an array using a loading blade followed by filling with immersion fluid and sealed. The PCR thermocycling reaction was performed in a QS3D PCR system (Life Technologies, Chicago, USA) with the following conditions: one cycle of 96 °C for 10 min, 40 cycles of 95 °C for 15 seconds followed by 60 °C for 1 min and a final extension cycle at 60 °C for 5 min. Following PCR thermocycling, QS3D digital PCR chips were read in a QS3D Microchip reader and data were analyzed in QS3D analysis suite software (Life Technologies, Chicago, USA) to quantitate RPP30 copies per sample (cps). Since two copies of RPP30 genomic sequences are present in a single diploid human cell. The total cell count in a sample can be calculated as half of the total number of RPP30 copies per sample. The total number of RPP30 copies quantitated in a ddPCR assay was used to determine the total number of RPP30 copies per sample using the following equation: Total cell counts in a sample equal to

$$\frac{[Total\,gDNA(ng)\,in\,a\,Sample]\times [RPP30\,cps\,in\,gDNA(ng)\,Template]\,\times [Dil.\,Factor]}{gDNA(ng)\,used\,in\,ddPCR\,assay\times 2}$$

Precision and accuracy determination experiments were carried out with CEPH 1347-02 human female human gDNA reference standard (CAT# 403062, Life Technologies, Chicago, USA) and RM gDNA reference standard (CAT# D1534999 G-01, Biochain Institute Inc, USA) containing 15000 RPP30 copies in 50000 pg was used as control gDNA. gDNA extracted from mouse derived SVEC 4-10 LECs served as negative control. RPP30 ddPCR assay precision and accuracy determination experiments were performed with human genomic DNA reference standard (Life Technologies, Chicago, USA) and genomic DNA extracted from RMs serially diluted from 100000 to 100 pg used as assay templates to amplify RPP30 genomic sequences. Observed RPP30 copies in the ddPCR assay were correlated with nominal RPP30 copies present in human and RM reference standard.

### Real-Time PCR (qPCR)

Real time PCR or qPCR reactions were performed in a 20 µL reaction mixture containing 10 µL Taqman Gene expression master mix (Life Technologies Inc, Chicago, IL), 1 µL of 20× custom RPP30 Taqman gene expression assay using above described primers and VIC labeled probe, 100 to 100000 pg of gDNA template in separate reactions and nuclease free water to make up the total reaction volume. Thermocycling was performed in C1000 with detection system CFX96 (Biorad, Los Angeles, CA) with the thermocycling conditions described above.

### Statistical analysis

Correlation analyses were performed between PBMCs, LNMCs and RMCs counts measured by automated cytometer and RPP30 copy number based cell counts in Prism software (Graph Pad Software Inc, La Jolla, USA). Pearson correlation coefficient (R^2^ value) and p values were calculated for PBMCs, LNMCs, RMCs and all PBMC and TIMC cell counts measured by automated cytometer and counts obtained from the RPP30 ddPCR assay. Linear regression (Prism software) of the amount of gDNA template in pg and the total number of RPP30 copies was used to assess RPP30 ddPCR assay precision. Coefficient of variation (%CV) was determined for the RPP30 copies quantitated in three replicates at each tested human and RM gDNA template concentration ranging from 100–100000 pg using the mean of RPP30 copy numbers obtained in three replicates after end point measurements in the ddPCR assay. In the qPCR assays, the mean of linearized C_T_ (2^−CT^) values of triplicates was used to correlate with human and RM gDNA template concentration. The accuracy of qPCR was analyzed by correlating RPP30 copies quantitated in the ddPCR assay versus known RPP30 copies of a reference human or RM standard at tested concentration as described previously^18^. Residual plots associated with linear regression showed the distance of various replicates from the curve. For drug normalizations, the concentration of drugs were determined by mass spectrometric analysis and represented as fM per a million cells (PBMCs, LNMCs and RMCs). Statistical significance analysis was performed between two groups using a student t test.

## Electronic supplementary material

Supplemental Information
