# Supplementary material for: Normalization of cell associated antiretroviral drug concentrations with a novel RPP30 droplet digital PCR assay
Source: Sci Rep. 2018 Feb 26;8:3626. doi: 10.1038/s41598-018-21882-0 (PMC5827666; doi:10.1038/s41598-018-21882-0)
Supplement: Supplementary file 1 — Supplemental Information [file 41598_2018_21882_MOESM1_ESM.pdf]

Supplementary Information

Normalization of cell associated antiretroviral drug concentrations with a novel RPP30 droplet digital PCR assay

Shetty Ravi Dyavar [a*], Zhen Ye [b], Siddappa N. Byrareddy [c], Kimberly K. Scarsi [a], Lee C. Winchester [a], Jonathan A. Weinhold [a], Courtney V. Fletcher [a*], Anthony T. Podany [a*]

[a] Antiviral Pharmacology Laboratory, Department of Pharmacy Practice, College of Pharmacy (COP), University of Nebraska Medical Center (UNMC), Omaha, NE 68198 USA

[b] Department of Pharmaceutical Sciences, COP, UNMC, Omaha, NE 68198 USA

[c] Department of Pharmacology and Experimental Neuroscience, College of Medicine, UNMC, Omaha, NE 68198, USA.

*Correspondence: shettyravi.dyavar@unmc.edu, apodany@unmc.edu and cfletcher@unmc.edu

Supplementary Methods

Sequence Alignment of RPP30 gene sequences

The RPP30 genomic sequence present at the NC_018921 locus on chromosome 10, alternate assembly CHM1_1.1 between 92909573 to 92909633 of human whole genome shot gun sequence (Accession no# NC_018921). This sequence homology among genomic DNA sequences of various NHP species and was selected with the multiple sequence alignment tool in The European Molecular Biology Laboratory at the European Bioinformatics Institute's Clustal Omega online program. The relationship between the sequences were represented as a hierarchical clustering tree.

Cell Associated Drug Quantitation in lysed TIMCs

TIMCs were sonicated for three rounds of 10 second cycles at 15 amplitude and followed by three 10 second cycles at 35 amplitude with 5 second intervals on ice in a sonicator (Q Sonicators, LLC, Newton, CT) apparatus to lyse the cells. Tenofovir-diphosphate (TFV-DP) and emtricitabine-triphosphate (FTC-TP) as previously described (1-3).

Supplementary Figures

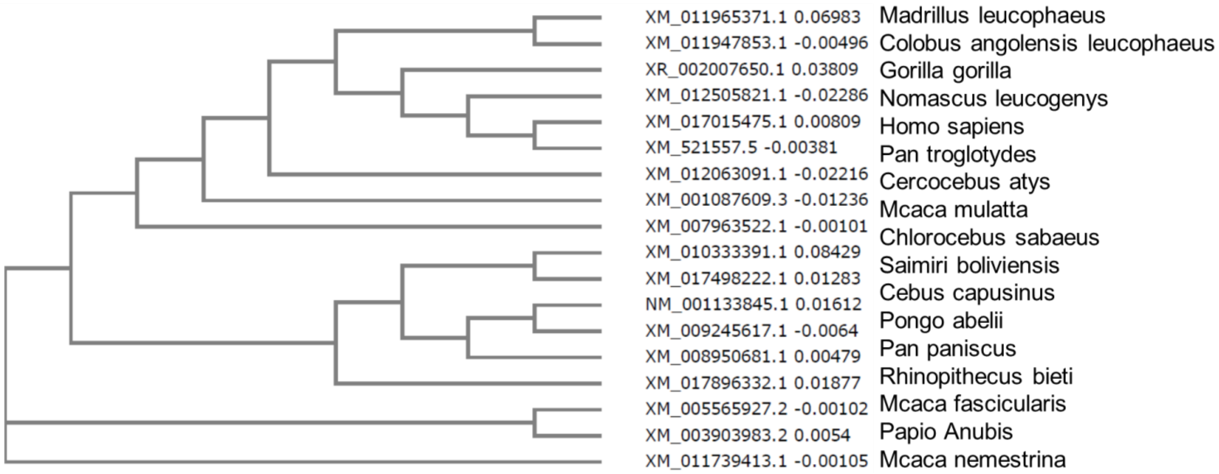

Figure S1. RPP30 genomic target sequence homology and clustering among human and non-human primate (NHP) species using Clustal Omega online program.

	FP PROBE RP
Homo sapiens	AGATTTGGACCTGCGAGCGGGTTCTGACCTGAAGGCTCTGCGCGGACTTGTGGAGACAGCCGCTC
Pan troglodytes	AGATTTGGACCTGCGAGCGGGTTCTGACCTGAAGGCTCTGCGCGGACTTGTGGAGACAGCCGCTC
Gorilla gorilla	AGATTTGGACCTGCGAGCGGGTTCTGACCTGAAGGCTCTGCGCGGACTTGTGGAGACAGCCGCTC
Nomascus leucogenys	AGATTTGGACCTGCGAGCGGGTTCTGACCTGAAGGCTCTGCGCGGACTTGTGGAGACAGCCGCTC
Pan paniscus	AGATTTGGACCTGCGAGCGGGTTCTGACCTGAAGGCTCTGCGCGGACTTGTGGAGACAGCCGCTC
Rhinopithecus roxellana	AGATTTGGACGTGCGAGCGGGTTCTGACCTGAAGGCTCTGCGCGGACTTGTGGAGACAGCGGCTC
Rhinopithecus bieti	AGATTTGGACGTGCGGGCGGGTTCTGACCTGAAGGCTCTGCGCGGACTTGTGGAGACAGCGGCTC
Macaca fascicularis	AGACTTGGACGTGCGAGCGGGTTCTGACCTGAAGGCTCTGCGCGGACTTGTGGAGACAGCGGCTC
Macaca mulatta	AGACTTGGACGTGCGAGCGGGTTCTGACCTGAAGGCTCTGCGCGGACTTGTGGAGACAGCGGCTC
Macaca nemestrina	AGACTTGGACGTGCGAGCGGGTTCTGACCTGAAGGCTCTGCGCGGACTTGTGGAGACAGCGGCTC
Mandrillus leucophaeus	AGACTTGGACGTGCGAGCGGGTTCTGACCTGAAGGCTCTGCGCGGACTTGTGGAGACAGCGGCTC

Figure S2. Clustal Omega multiple sequence alignment analysis of RPP30 amplicon used by Luo et al study (doi: 10.1373/clinchem.2013.206375) with genomic sequences in humans and non-human primate species. Forward primer (FP, green), reverse primer (RP, dark red) and probe (blue) conserved sequences and non-homologous mutations (orange) in various non-human primate species were shown.

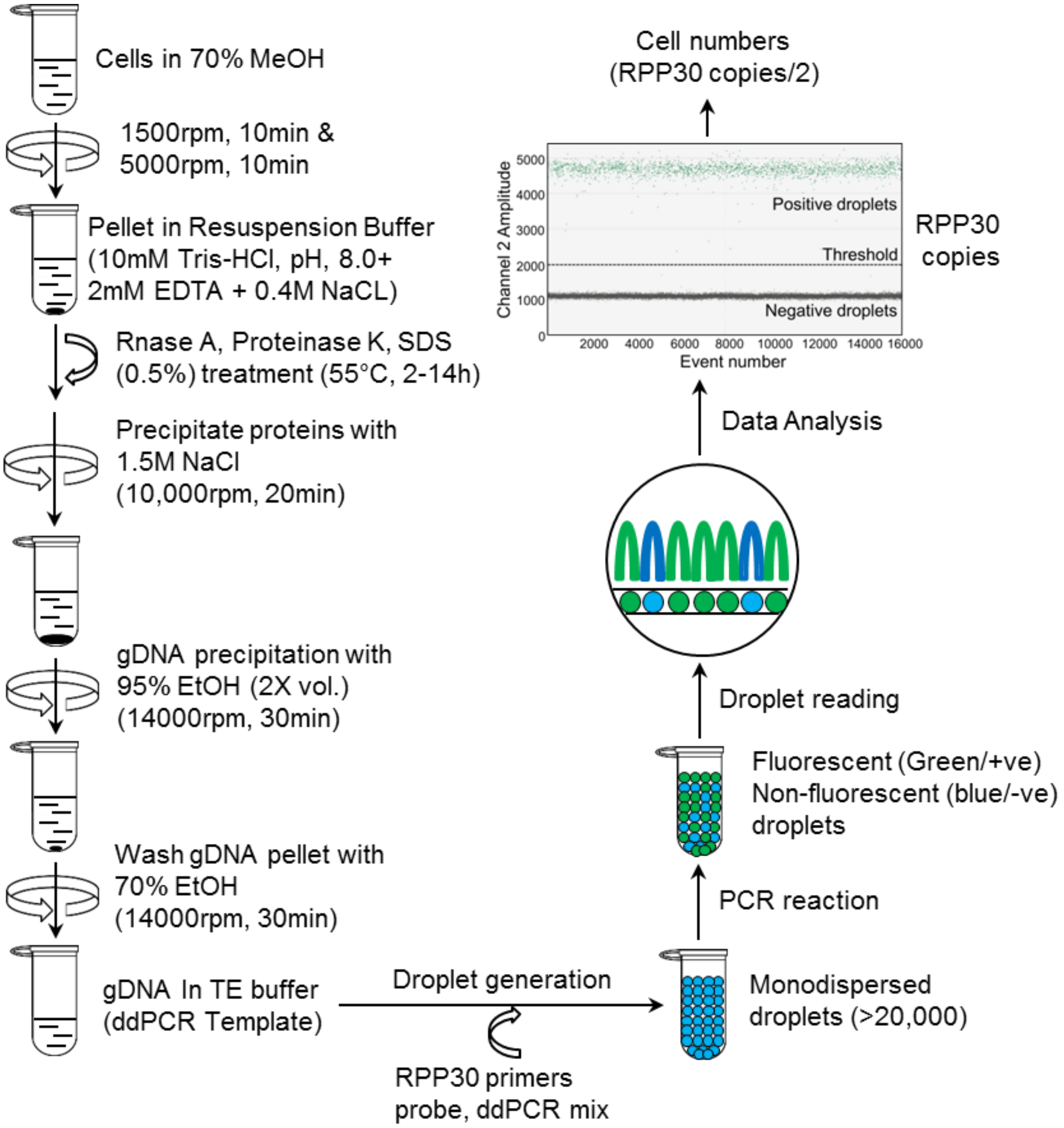

Figure S3. Schematic diagram of gDNA extraction from intracellular drug samples (tissue isolated from mononuclear cells in 70% methanol) and RPP30 ddPCR assay work flow using gDNA templates to derive cell counts. PBMCs, peripheral blood mononuclear cells and TIMCs, tissue isolated mononuclear cells.

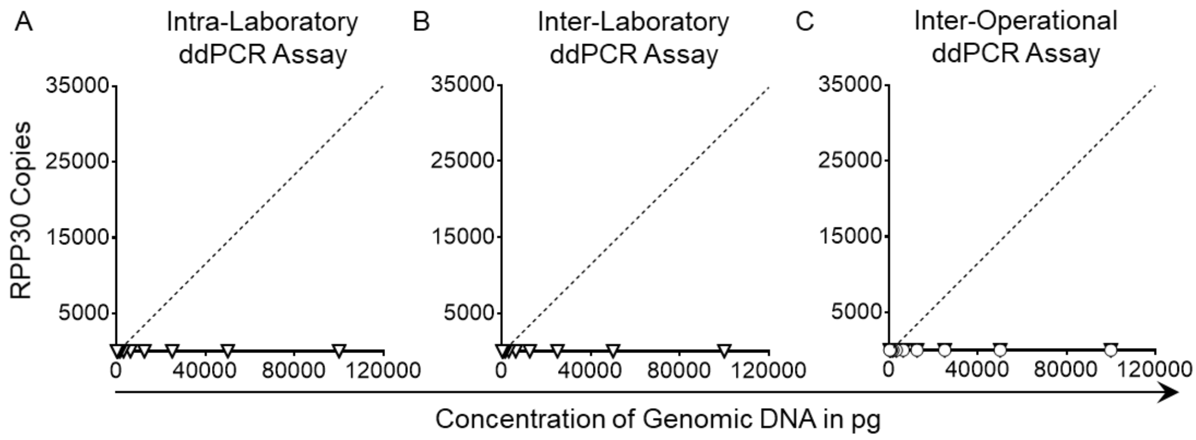

Figure S4. Precision of human and rhesus macaque RPP30 ddPCR assay. RPP30 copy number quantitated in two fold dilution of 100000pg of mouse gDNA in intra-laboratory (A), inter-laboratory (B) and inter-operational (C) assays. Standard curve for quantifying RPP30 copies in 2 fold dilutions (100-100000pg). RPP30copies on the y axis and conc. Of gDNA in pg on the x axis.

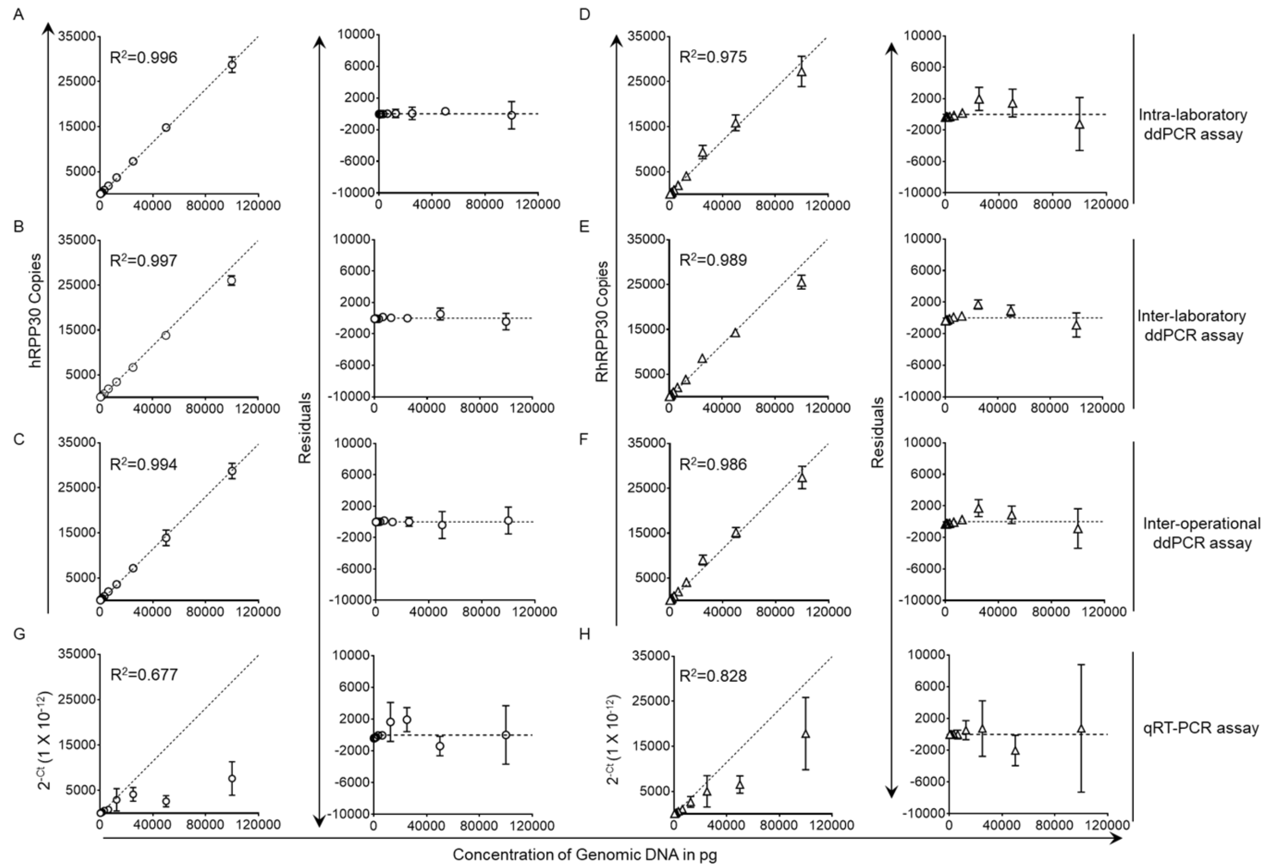

Figure S5. Precision of human and rhesus macaque RPP30 ddPCR and qPCR assays. RPP30 copy number quantitated in two fold increase in template conc. from 100-100000pg using human gDNA reference standard containing 15000 RPP30 copies per 50000pg in intra-laboratory (A), inter-laboratory (B) and inter-operational (C) ddPCR assays and in intra-qPCR assays (G); rhesus macaque gDNA reference standard in intra-laboratory (D), inter-laboratory (E) and inter-operational (F) ddPCR assays and intra-human (G) and RM (H) qPCR assays. Standard curve for quantifying RPP30 copies in two fold increase in gDNA template conc. from 100-100000pg and linear regression analysis was performed between RPP30copies on the y axis and conc. of gDNA on the x axis for the plots on the left and residuals of the plotted values were shown on the plots on the right.

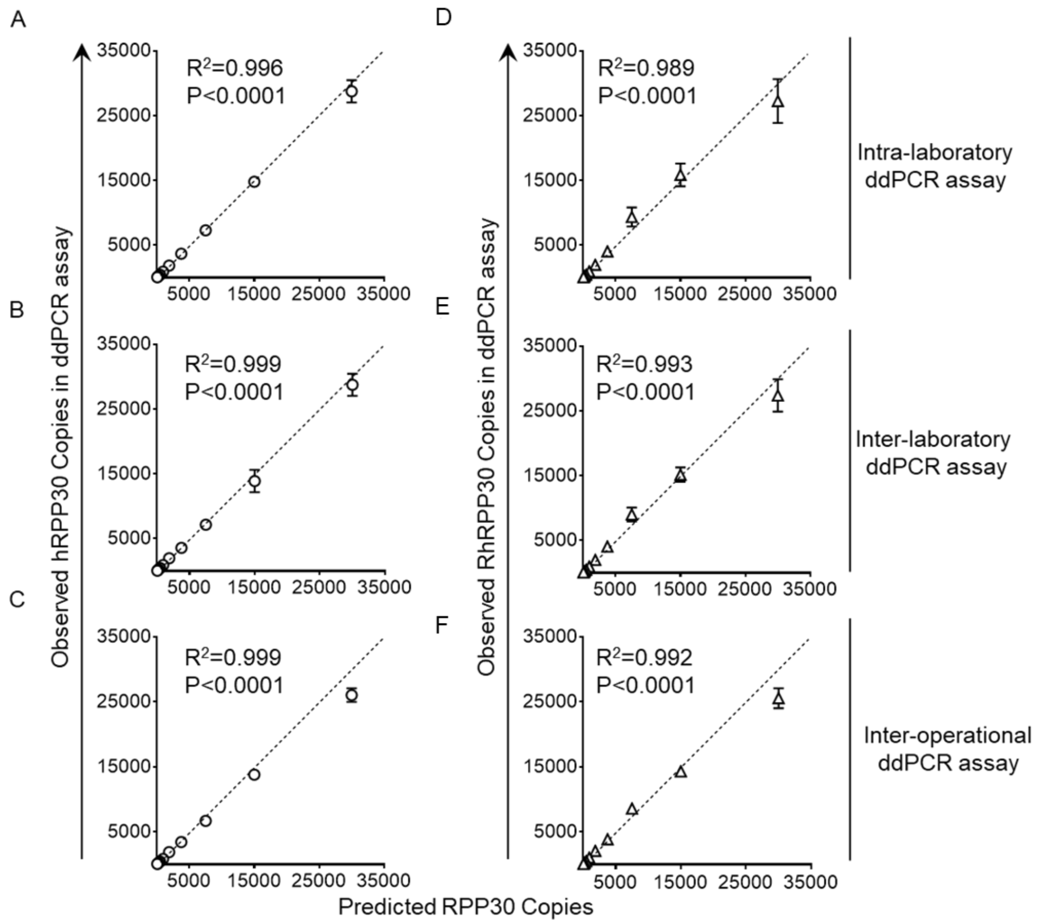

Figure S6. Accuracy of RPP30 ddPCR assay. RPP30 copy numbers were quantitated in a two-fold increments of human and rhesus macaque gDNA concentration from 100 to 100000pg. Human gDNA reference standard containing 15000 RPP30 copies per 50000pg of gDNA was used as a reference of nominal RPP30 copies in ddPCR assays. Observed RPP30 copies in ddPCR assays were correlated with nominal RPP30 copies based on the reference standards in human gDNA templates in intra-laboratory (A), inter-laboratory (B) and inter-operational (C) assays; and in rhesus macaque gDNA templates in intra-laboratory (D), inter-laboratory (E) and inter-operational (F) assays. Correlation curve for observed and nominal RPP30 copies in two fold increase of gDNA template conc. from 100-100000pg were plotted on the y and x axis respectively.

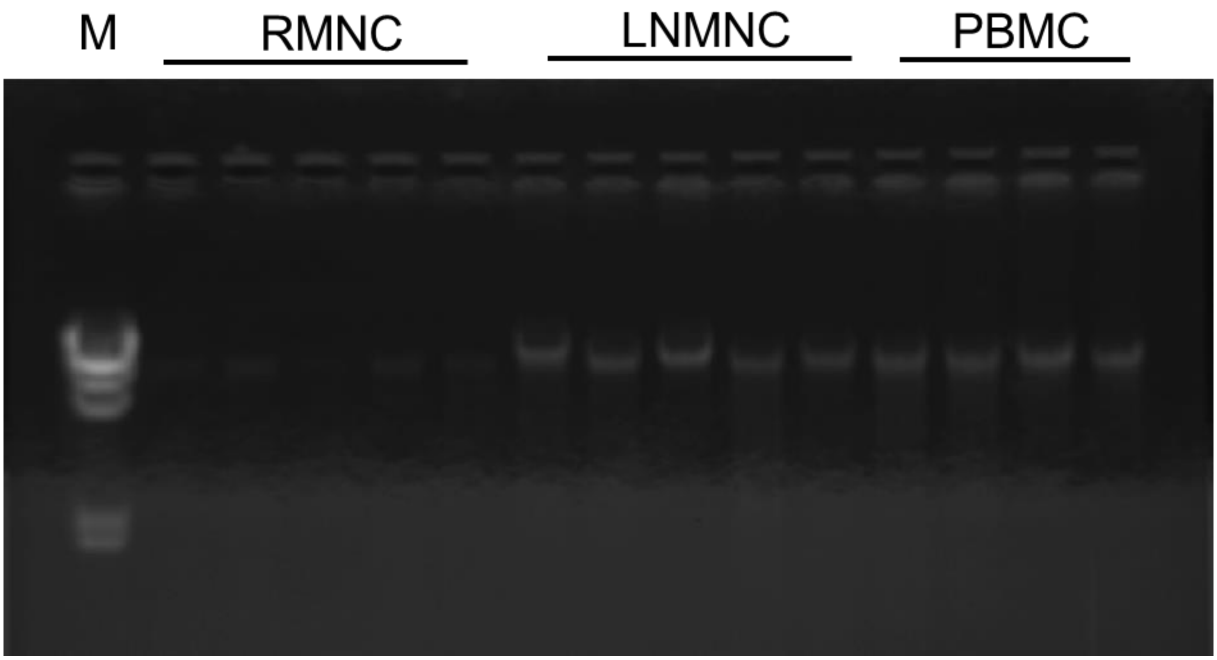

Figure S7. Qualitative analysis of genomic DNA extracted from mononuclear cells isolated from rectum (RMNCs), lymph node (LNMNCs) and peripheral blood (PBMCs) tissues of ART patients on one percent agarose gel. M, molecular marker; RMNC, rectum derived mononuclear cells; LNMNC, lymph node derived mononuclear cells and PBMC, peripheral blood mononuclear cells.

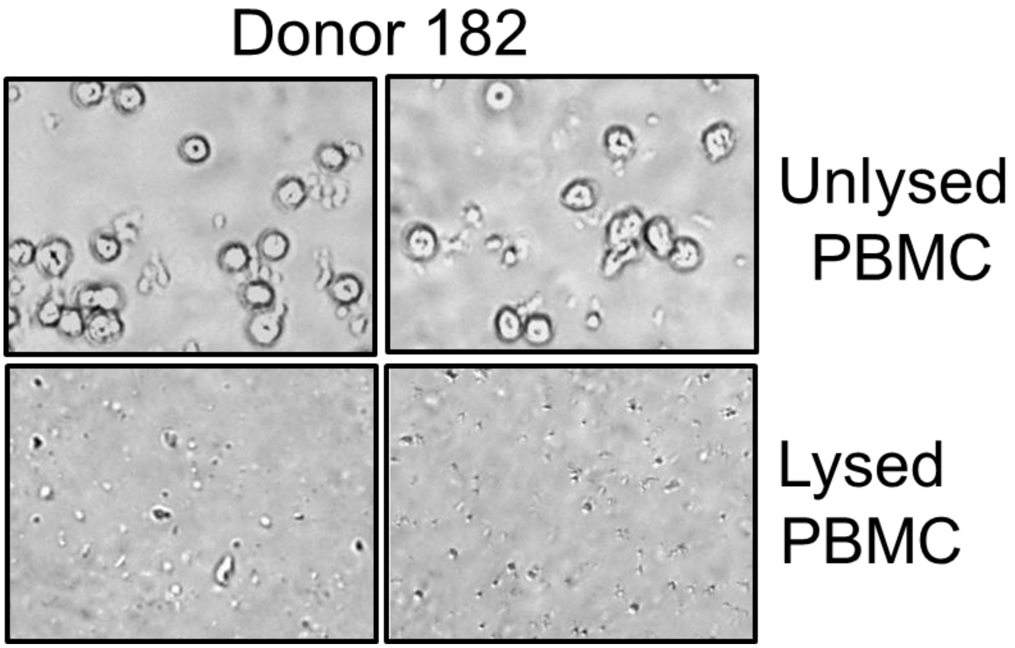

Figure S8. EVOS fluid cell microscopic images of lysed and unlysed peripheral blood mononuclear cells (PBMC) of ART patients.

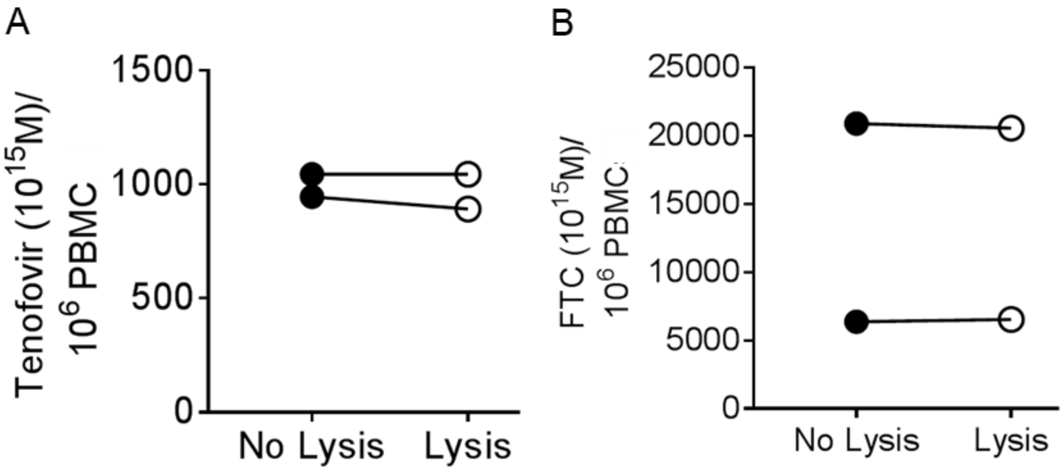

Figure S9. Quantity of A) Tenofovir (TFV) and B) Emtricitabine (FTC) antiretroviral drugs in unlysed (intact) and lysed (destructed by sonication) PBMCs clinical samples measured by Mass spectrometry.

Table S1. Precision of human RPP30 ddPCR assays. A) Mean, standard deviation (STD DEV) and %CV of intra (n=3) and inter-human RPP30 ddPCR assays (n=8) are shown

gDNA conc.	Intra-RPP30 ddPCR Assay				Inter-RPP30 ddPCR Assay			
	Mean	STD DEV	%CV	n	Mean	STD DEV	%CV	n
100000	28755.7	1730.1	6.0	3	28738.5	1709.6	5.9	8
50000	14816.3	394.2	2.7	3	13897.1	758.8	5.5	9
25000	7314.9	795.0	10.9	3	7163.6	575.3	8.0	9
12500	3706.4	529.2	14.3	3	3578.4	286.9	8.0	9
6250	1870.7	175.0	9.4	3	1896.4	175.0	9.2	9
3125	919.8	85.6	9.3	3	919.8	85.6	9.3	9
1573	453.0	33.1	7.3	3	453.0	33.1	7.3	9
781	241.7	39.4	16.3	3	254.1	35.6	14.0	9
391	129.9	12.5	9.6	3	129.9	6.7	5.2	9
195	70.1	3.8	5.4	3	70.1	3.8	5.4	9
98	35.6	1.4	4.0	3	39.9	4.3	10.8	9

Table S2. Precision of rhesus macaque RPP30 ddPCR assays. Mean, standard deviation (STD DEV) and %CV of intra (n=3) and inter (n=9) rhesus macaque RPP30 ddPCR assays are shown.

gDNA conc.	Intra-RPP30 ddPCR Assay				Inter-RPP30 ddPCR Assay			
	Mean	STD DEV	%CV	n	Mean	STD DEV	%CV	n
100000	27253.7	3377.8	12.4	3	27253.7	2507.3	9.2	9
50000	15857.5	1764.8	11.1	3	15161.3	1112.8	7.3	9
25000	9361.1	1477.4	15.8	3	9017.0	1076.2	11.9	9
12500	4048.6	420.4	10.4	3	4048.6	420.4	10.4	9
6250	1983.3	131.3	6.6	3	1990.1	144.3	7.2	9
3125	952.8	40.3	4.2	3	976.6	51.3	5.3	9
1573	463.4	2.7	0.6	3	491.8	54.1	11.0	9
781	265.2	16.6	6.3	3	272.9	16.6	6.1	9
391	132.2	15.8	12.0	3	144.9	15.8	10.9	9
195	68.2	3.2	4.6	3	73.6	5.2	7.1	9
98	48.1	7.6	15.8	3	38.8	2.4	6.2	9

Table S3. Precision of rhesus macaque RPP30 ddPCR assays. Mean of linearized CT (2^{-CT}) values, STD DEV and %CV of human (n=3) and rhesus macaque (n=3) RPP30 qPCR assays are shown.

gDNA conc.	Human-RPP30 ddPCR Assay				RM-RPP30 ddPCR Assay			
	Mean (1×10^{12})	STD DEV	%CV	n	Mean (1×10^{12})	STD DEV	%CV	n
100000	7667.1	3681.3	48.0	3	17823.1	8023.3	45.0	3
50000	2652.4	1216.7	45.9	3	6508.4	1919.9	29.5	3
25000	4163.3	1516.0	36.4	3	5019.9	3502.5	69.8	3
12500	2966.2	2459.1	82.9	3	2669.5	1200.0	45.0	3
6250	829.7	416.5	50.2	3	1061.6	549.2	51.7	3
3125	597.9	370.2	61.9	3	601.4	239.0	39.7	3
1573	264.1	143.3	54.3	3	294.2	145.0	49.3	3
781	143.4	78.6	54.8	3	124.9	36.1	28.9	3
391	90.2	27.3	30.2	3	73.6	40.4	54.8	3
195	31.3	14.9	47.6	3	27.9	18.0	64.8	3
98	17.9	12.7	70.8	3	9.2	5.5	60.2	3

Table S4. Standards for Reporting Diagnostic accuracy of RPP30 ddPCR assay.

Section & Topic	No	Item	Reported on page #
**TITLE OR ABSTRACT**			
	**1**	Identification as a study of diagnostic accuracy using at least one measure of accuracy<br>(such as sensitivity, specificity, predictive values, or AUC)	8-10
**ABSTRACT**			
	**2**	Structured summary of study design, methods, results, and conclusions<br>(for specific guidance, see STARD for Abstracts)	2
**INTRODUCTION**			
	**3**	Scientific and clinical background, including the intended use and clinical role of the index test	4-7
	**4**	Study objectives and hypotheses	7 and 16
**METHODS**			
Study design	**5**	Whether data collection was planned before the index test and reference standard were performed (prospective study) or after (retrospective study)	21
Participants	**6**	Eligibility criteria	20-21
	**7**	On what basis potentially eligible participants were identified<br>(such as symptoms, results from previous tests, inclusion in registry)	21
	**8**	Where and when potentially eligible participants were identified (setting, location and dates)	21
	**9**	Whether participants formed a consecutive, random or convenience series	NA
Test methods	**10a**	Index test, in sufficient detail to allow replication	25-28
	**10b**	Reference standard, in sufficient detail to allow replication	28
	**11**	Rationale for choosing the reference standard (if alternatives exist)	28
	**12a**	Definition of and rationale for test positivity cut-offs or result categories of the index test, distinguishing pre-specified from exploratory	14
	**12b**	Definition of and rationale for test positivity cut-offs or result categories of the reference standard, distinguishing pre-specified from exploratory	9-11

	**13a**	Whether clinical information and reference standard results were available to the performers/readers of the index test	21, 22
	**13b**	Whether clinical information and index test results were available to the assessors of the reference standard	14, 15
Analysis	**14**	Methods for estimating or comparing measures of diagnostic accuracy	26-28
	**15**	How indeterminate index test or reference standard results were handled	29, 30
	**16**	How missing data on the index test and reference standard were handled	NA
	**17**	Any analyses of variability in diagnostic accuracy, distinguishing pre-specified from exploratory	8-10
	**18**	Intended sample size and how it was determined	14, 15
**RESULTS**			
Participants	**19**	Flow of participants, using a diagram	NA
	**20**	Baseline demographic and clinical characteristics of participants	NA
	**21a**	Distribution of severity of disease in those with the target condition	NA
	**21b**	Distribution of alternative diagnoses in those without the target condition	NA
	**22**	Time interval and any clinical interventions between index test and reference standard	NA
Test results	**23**	Cross tabulation of the index test results (or their distribution) by the results of the reference standard	8-11
	**24**	Estimates of diagnostic accuracy and their precision (such as 95% confidence intervals)	8-11
	**25**	Any adverse events from performing the index test or the reference standard	NA
**DISCUSSION**			
	**26**	Study limitations, including sources of potential bias, statistical uncertainty, and generalisability	NA
	**27**	Implications for practice, including the intended use and clinical role of the index test	19-20
**OTHER INFORMATION**			
	**28**	Registration number and name of registry	NA
	**29**	Where the full study protocol can be accessed	NA
	**30**	Sources of funding and other support; role of funders	37

Supplementary References

1) Delahunty, T., Bushman, L., Robbins, B. & Fletcher, C. V. The simultaneous assay of tenofovir and emtricitabine in plasma using LC/MS/MS and isotopically labeled internal standards. *Journal of Chromatography B* **877**, 1907–1914 (2009).
2) King, T. *et al*. Liquid chromatography–tandem mass spectrometric determination of tenofovir-diphosphate in human peripheral blood mononuclear cells. *Journal of chromatography B* **843**, 147–156 (2006).
3) Fletcher, C. V. *et al*. Persistent HIV-1 replication is associated with lower antiretroviral drug concentrations in lymphatic tissues. *Proceedings of the National Academy of Sciences* **111**, 2307–2312 (2014).
